# The Conserved *Candida albicans* CA3427 Gene Product Defines a New Family of Proteins Exhibiting the Generic Periplasmic Binding Protein Structural Fold

**DOI:** 10.1371/journal.pone.0018528

**Published:** 2011-04-11

**Authors:** Sébastien Santini, Jean-Michel Claverie, Nicolas Mouz, Tristan Rousselle, Caroline Maza, Vincent Monchois, Chantal Abergel

**Affiliations:** 1 Information Génomique et Structurale (CNRS UPR2589), Aix-Marseille Université, Mediterranean Institute of Microbiology, Parc Scientifique de Luminy, Marseille, France; 2 PX'Therapeutics - 7 Parvis Louis Néel – BP50, Grenoble, France; Deutsches Krebsforschungszentrum, Germany

## Abstract

Nosocomial diseases due to *Candida albicans* infections are in constant rise in hospitals, where they cause serious complications to already fragile intensive care patients. Antifungal drug resistance is fast becoming a serious issue due to the emergence of strains resistant to currently available antifungal agents. Thus the urgency to identify new potential protein targets, the function and structure of which may guide the development of new antifungal drugs. In this context, we initiated a comparative genomics study in search of promising protein coding genes among the most conserved ones in reference fungal genomes. The CA3427 gene was selected on the basis of its presence among pathogenic fungi contrasting with its absence in the non pathogenic *Saccharomyces cerevisiae*. We report the crystal 3D-structure of the *Candida albicans* CA3427 protein at 2.1 Å resolution. The combined analysis of its sequence and structure reveals a structural fold originally associated with periplasmic binding proteins. The CA3427 structure highlights a binding site located between the two protein domains, corresponding to a sequence segment conserved among fungi. Two crystal forms of CA3427 were found, suggesting that the presence or absence of a ligand at the proposed binding site might trigger a “Venus flytrap” motion, coupled to the previously described activity of bacterial periplasmic binding proteins. The conserved binding site defines a new subfamily of periplasmic binding proteins also found in many bacteria of the bacteroidetes division, in a choanoflagellate (a free-living unicellular and colonial flagellate eukaryote) and in a placozoan (the closest multicellular relative of animals). A phylogenetic analysis suggests that this gene family originated in bacteria before its horizontal transfer to an ancestral eukaryote prior to the radiation of fungi. It was then lost by the Saccharomycetales which include *Saccharomyces cerevisiae*.

## Introduction

*Candida* spp are ubiquitous commensal organisms that can cause serious disseminated infections, particularly in immunocompromised and intensive care patients. *Candida* spp. are the fourth leading cause of nosocomial bloodstream infections in the United States, with treatment costs estimated to be more than $2–$4 billion annually [1] and with mortality rates estimated between 38% to 49% [2]. Candidiasis is the most common invasive fungal infection reported in cancer patients (58%–69%) [3], and over the past decade, the incidence of these fungal infections has increased significantly [4]. Although resistance to antifungal drugs remains uncommon on community acquired infections, they are in constant rise in nosocomial infections [5]. Since it has been demonstrated that clinical isolates of the *Candida* species *C. albicans*, *C. glabrata*, *C. tropicalis*, and *C. krusei* have acquired resistance against first-line agents for treatment of invasive candidiasis by mutations in the gene encoding the target enzyme (glucan synthase) [5], [6], [7], it appears important to anticipate and enlarge the antifungal drug spectrum by identifying new original targets. In this context, our laboratory led a prospective structural genomics project (PROFUN [8]) in search of new antifungal targets.

The gene selection was based on the comparison of the following fungi genomes: *Candida albicans* (SC5314), *Saccharomyces cerevisiae* (S288C), *Neurospora crassa* (OR74A), *Magnaporthe grisea* (70-15 (Mat1-1)), *Schizosaccharomyces pombe*, *Aspergillus fumigatus* (Af293), *Phanerochaete chrysosporium*, *Cryptococcus neoformans* ((Serotype D) JEC21+B3501), *Cryptococcus neoformans* ((Serotype A) H99). This study aimed to identify virulence-related targets by focusing on genes conserved in pathogenic fungi and absent from the Saccharomyces *cerevisiae* genome. The *CA3427* gene belongs to this category and encodes a 299 amino acid-long, 33.7 kDa molecular weight protein of unknown function (UNIPROT: Q59X88).

The comparison of the CA3427 sequence with its database homologs clearly highlights a new functional family conserved (>30% identity over its entire lenght) across most fungi genomes and present in some flavobacteria. It only shares a weak similarity (<20% identity over the full length sequences) with the Pyrimidine precursor biosynthesis THI13 enzyme from *S. cerevisiae*. To gain insights into the function and druggability of CA3427 we determined its crystal structure by the multi-wavelength anomalous dispersion (MAD) method [9]. Interestingly, two crystal forms were obtained which seem to correspond to a large conformational change induced by the binding of a small ligand at a specific site of the protein.

## Methods

### Cloning expression and purification

As part of the larger structural genomics PROFUN project, CA3427 was produced using the protocol previously described for other targets [10]. Briefly, the cDNA was isolated by PCR using sequence primers specific to the CA3427 gene preceded by 5′-CATCACCATCAATTG (Direct primer) and 5′-TCACCATCCAATTG (Reverse Primer) applied to a template of purified genomic DNA from the *Candida albicans* strain NIH 3147 (ATCC number MYA-2876D). Gene cloning was performed using the ligation-independent cloning (LIC) method and our pSF-04 expression vector [10]. The PCR products were directly purified using the NucleoSpin Extract kit (Macherey Nagel). Then, 0.2 pmol of the purified PCR product was treated with T4 DNA polymerase in the presence of 2.5 mM of dCTP for 30 minutes at 22°C before inactivating the enzyme (20 minutes at 75°C). In a parallel procedure, the pSF-04 expression vector was digested with the *Mfe*l restriction enzyme to excise the insert bearing the lacZ encoding sequence. pSF-04 was then purified on agarose gel using the NucleoSpin Extract kit (Macherey Nagel) and treated with T4 DNA polymerase in the presence of 2.5 mM of dGTP for 30 minutes at 22°C before inactivating the enzyme (20 minutes at 75°C).

The CA3427 cloning was performed as follows. A hybridization reaction was carried out by mixing 0.01 pmol of pSF-04 and 0.02 pmol of the insert in a reaction volume of 3 µl, followed by a 5 minutes incubation at 22°C and the subsequent addition of 1 µl of 25 mM EDTA. After a second incubation of 5 minutes at 22°C, the resulting product was used to transform *E. coli* DH5α. Transformants were selected on LB plates containing 100 µg/ml ampicillin, and positive colonies were isolated. This cloning procedure allowed the addition of a (His)_6_ tag followed by the GHHHQL sequence to the N-terminal of the CA3427 gene product and of a C-terminal QLDGDLEAA linker to the GFP protein.

An expression screen was then performed using our standard procedure [11]. The GFP reporter was used to quantify (and determine the optimal condition for) the soluble expression of the CA3427 protein through fluorescence measurements [12]. The subsequent removal of the GFP-encoding gene was done by *Not*I digestion followed by the circularization of the plasmid.

The plasmid born CA3427 gene was over-expressed in *E. coli* BL21 in 1L flasks containing TB medium over one night at 17°C after induction with IPTG (500 µM) at OD_600 nm_ = 0.5. The selenomethionine-substituted protein was produced using the appropriate protocol to inhibit methionine synthesis in the presence of selenomethionine and M9 minimal medium [13].

After centrifugation, the pellet was resuspended in buffer A (50 mM NaH_2_PO_4_, 300 mM NaCl pH 8.0) with 5% glycerol and 0.1% Triton X-100 then sonicated and centrifuged again.

The cleared lysate was applied to a 5 ml HiTrap Chelating Column (GE Healthcare) charged with Ni^2+^ and equilibrated with buffer A. The column was washed with 10 column volumes of buffer A, 10 column volumes of buffer A containing 25 mM Imidazole and 5 column volumes of buffer A containing 50 mM Imidazole at a flow rate of 1 ml.min^−1^. Elution was performed with a linear gradient over 7 column volumes from 50 mM to 500 mM Imidazole. The fractions corresponding to the elution of CA3427 with 150–200 mM Imidazole were run on a desalting column (Fast Desalting Column HR 10/10, Pharmacia) and we controlled the recombinant protein sequence by mass spectroscopy and N-terminal Edman sequencing. After purification, the fractions contained at least 98% pure protein in 10 mM Tris buffer at pH 7. Upon isoelectric focusing chromatography, the recombinant CA3427 protein showed a PI≈5. The analysis by dynamic light scattering of the purified recombinant CA3427 protein indicated a monodisperse solution with a gyration radius of ≈2.5 nm, compatible with a monomer.

### Crystallization

The *C. albicans* CA3427 recombinant protein was concentrated to 18.5 g/L in 10 mM MOPS buffer at pH 7.5 using a centrifugal filter device (Ultrafree Biomax 10K, Millipore, Bedford MA, USA). The screening for crystallization conditions was performed using a standard strategy [10].

The best crystals were obtained using the hanging drop vapor diffusion method with a 1 ml reservoir. Crystallization droplets were made of 0.5 µl of protein mixed with 0.5 µl of the reservoir solution made of 21% PEG8000, 0.2 M Calcium Acetate, 0.1 M Tris, 30% Glycerol at a pH of 7.0 (structure 1) and 13% PEG8000, 0.2 M Calcium Acetate, 0.1 M Tris, 10% Glycerol at a pH of 7.0 (structure 2). Crystals appeared within a few days.

To explore the CA3427 specificity, we performed co-crystallization experiments with a variety of ligands at a concentration of 1 mM (pyridoxal phosphate, histidine, lysine, arginine, Glutamine, Leucine, Isoleucine). None of them resulted in a liganded structure with extra electron density in the CA3427 binding site.

### Data collection

Crystals of the CA3427 protein were mounted in a Hampton Research 0.2 mm^3^ loop, flash frozen to 100K in a cold nitrogen gas stream and subjected to X-rays. The two datasets were collected on a MarCCD (165 mm) camera at the European Synchrotron Radiation Facility (ESRF) on the BM30A-FIP beamline.

The first *C. albicans* CA3427 structure (PDB: 2X7P) was determined using the MAD method based on a two-wavelenght data set (Table 1) obtained with a selenomethionine-substituted protein crystal. The crystals belong to the orthorhombic space group P2_1_2_1_2_1_ with unit cell parameters a = 42.588 Å, b = 66.849 Å, c = 113.990 Å, α = β = γ = 90.

**Table 1:** X-ray data collection (ESRF) and refinement statistics.

Data collection	2X7P		2X7Q
Beam line	BM30A		ID29
Method	MAD		Molecular Replacement
Wavelength (Å)	0.979774	0.979958	0.975627
Space group	P 21 21 21		P 21 21 21
Unit cell (Å)	a = 42.588 b = 66.849 c = 113.990 α = 90 β = 90 γ = 90		a = 41.411 b = 65.724 c = 128.203 α = 90 β = 90 γ = 90
Resolution range (Å)	43.337 to 2.341	43.337 to 2.341	46.127 to 2.0
(highest resolution shell)	(2.62 to 2.51)	(2.47o 2.34)	(2.11 to 2.0)
Observations	61514 (1615)	76662 (4193)	147779 (16688)
Unique reflections	10802 (534)	13379 (1175)	17895 (2314)
Multiplicity^1^	5.5 (2.4)	5.5 (3.0)	8.0 (6.8)
Completeness^1^	98.8 (89.6)	99.1 (94.4)	76.7 (72.6)
<I/σI>^1^^,^ 2	7.6 (3.9)	4.1 (3.0)	6.5 (2.7)
R_sym_ (%)^1^^,^ 3	6 (16.5)	7 (22.7)	9.6 (26.5)
**Refinement**			
R_cryst_ (%)^4^	20.9		21.8
R_free_ (%)	25.6		23.6
Δ_bond_ (Å)	0.001		0.002
Δ_angle_ (°)	0.394		0.478
N° Protein atoms	2463		2466
N° water	140		220
N° Heterogen atoms	46		8
**Average B factor (Å** 2 **)**			
All atoms	22.9		14.3
Protein main chain	22.2		13.7
Water	26		18.1
Ligand	28		18.8
**Ramachandran plot (%)**			
Most favored	261		258
Allowed	18		21
Generously allowed	0		0
Disallowed regions	1		1

1 values in parentheses are for the highest resolution shell.

2 <I/σ I>, is the mean signal to noise ratio, where I is the integrated intensity of a measured reflection and σ is the estimated error in the measurement.

3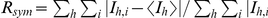, where I is the integrated intensity of reflection h having i observations and 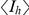 is the mean recorded intensity of reflection h over multiple recording.

4 R_cryst_ = 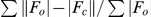, where F_o_ are observed and F_c_ calculated structure factor amplitudes. R_free_ is calculated from a randomly chosen 9.9% of reflections.

The second dataset (PDB: 2X7Q) was collected at a wavelength of 0.975627 Å. The crystals belong to the P2_1_2_1_2_1_ space group with unit cell parameters a = 41.411, b = 65.724, c = 128.203, α = β = γ = 90.

### Structures determination and refinement

The diffraction data were indexed with MOSFLM [14] and scaled with the SCALA [15] software from the CCP4 suite [16].

Phase determination was performed by using the SOLVE program [17] on two wavelengths corresponding to the peak (0.979774 Å) and the inflexion point (0.979958 Å) in the 43.437 to 2.341 Å resolution range. A single solution was found with a mean figure of merit of 0.4 for all the data between 35 and 2.5 Å. The phases obtained were improved by using autoSHARP [18]. The electron-density map was used to construct the main chain of the molecules by using COOT [19]. Refinement was performed using the Phenix software [20] including manual rebuilding and rigid body refinement followed by several cycles of positional refinement.

For the second crystal form, we used molecular replacement on the CaspR server [21] and the MAD-solved three-dimensional structure of CA3427 as template. The structure was refined using COOT and iterative steps of manual rebuilding and positional refinement using Phenix. PROCHECK [22] was used to assess the quality of the structures. All statistics are presented in Table 1.

The atomic coordinates and structure factors for the crystal structures of the CA3427 protein from *Candida albicans* are available in the RCSB Protein Data Bank under PDB id 2X7P and 2X7Q.

### Phylogenetic analysis

The evolutionary relationship of CA3427 with its homologs was assessed as follows. We searched for orthologous sequences against the 82 available reference fungi genomes [23]. All BLAST [24] searches were performed on the servers hosting the corresponding fungi genomes with default parameters: BROAD Institute Fungal Genome Initiative [25], Department of Energy Joint Genome Institute [26], National Center for Biotechnology Information [27], Resources for Fungal Comparative Genomics [28] and Fungal Genome Research website [29]. Only 60 species showed an unambiguous homolog which were used to build the phylogenetic tree and compare it with a reference tree. To collect a larger panel of homologous sequences, the CA3427 sequence was used as a seed for BLAST search against the Ref-Seq database (NCBI). Sequences of bacterial origin, all belonging to the bacteroidetes/Flavobacteria clade, were readily identified as best matching the CA3427 protein sequence (E value<10^−20^), as well the two additional unexpected homologs of eukaryotic origin, one from the choanoflagellate *Monosiga brevicollis* and the other one from the placozoan *Trichoplax adhaerens*. All those sequences turned out to respect the residue conservation previously identified for the fungal CA3427 homologs strengthening the definition of a new periplasmic binding protein (PBP)-like subfamily. We then selected 15 non redundant representatives of the fungi that were retained for a detailed phylogenetic analysis together with 9 bacterial sequences as well as the choanoflagellate and the placozoan sequences. To identify a suitable outgroup of PBP-related sequences for rooting purpose, we selected more divergent, yet highly significant (E value<10^−6^), bacterial homolog sequences from a cyanobacterium, a firmicute, a beta and an alpha proteobacterium, none of them sharing the new subfamily signature. To optimize the multiple alignment, proteins were truncated at the domain boundaries of the CA3427 sequence. This dataset was used to study the evolutionary relationship within this new PBP-like subfamily using the phylogeny.fr web server [30]. Details of the parameter used for the computation are provided in the figures legends.

## Results and Discussion

### Overall structure of CA3427

**Figure 1:**
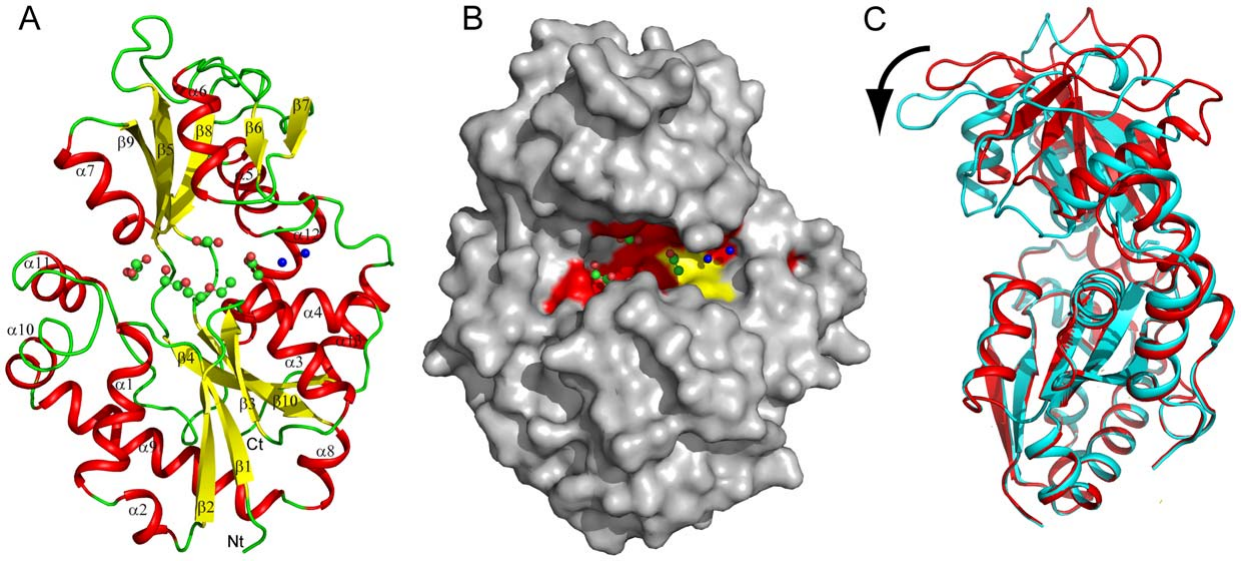
CA3427 structures. A) 2x7p is represented with alpha helices in red and beta-sheet in yellow. Secondary structures are numbered along the protein sequence. Carbon dioxide, glycerol and PEG molecules are in ball and sticks with green carbon and red oxygen atoms. 2 Water molecules are represented as blue spheres. B) Surface representation of the open conformation, 2x7p. The strictly conserved residues in the groove are colored in red and residues with conserved properties are marked in yellow. Carbon dioxide, glycerol and carboxyl molecules are in ball and sticks with green carbon and red oxygen atoms. Two water molecules are represented as blue spheres. C) The two conformations (2x7q: cyan, 2x7p: red) are superposed on domain I (bottom). The venus flytrap motion is illustrated by a black arrow (see Movie S1 for an animated view).

CA3427 is an α/β protein with two domains organized into a C-clamp shape (Fig. 1A–B). Domain I, encompassing residues 1 to 81 and 190 to 299, is composed of a 5 stranded β-sheet (β_2_ β_1_ β_3_ β_10_ β_4_) with β_10_ anti-parallel to the others, surrounded by 10 helices (α_1_ to α_4_ and α_8_ to α_13_). The smaller domain II, encompassing residues 88 to 183, is also arranged in a 5 stranded β-sheet (β_7_ β_6_ β_8_ β_5_ β_9_) with β_9_ anti-parallel to the others, surrounded by 3 α-helices (α_5_ α_6_ α_7_). The two domains delimit a large groove and are linked by a hinge region (residues 82–87 and 184–189).

### Comparison of the two CA3427 structures

The two crystal forms correspond to distinct conformations of the CA3427 protein with a root mean square deviation (RMSD) of 1.55 Å based on α-carbons superimposition of the overall structures. Most of the RMSD value results from a change in the relative position of the two domains rather than from local rearrangements (Fig. 1C, see Movie S1 for an animated view). This is well demonstrated by measuring the distances between three α-carbons delimiting the groove: E40, S140 and D236. The distances separating E40 from S140 varies from 12.44 Å to 8.41 Å between the two structures, and from 20.93 Å to 18.33 Å for the distance between D236 and S140.

To determine the motion best describing the transition between the two crystal structures, we performed a normal mode analysis on the El Némo server [31] using the two structures. The C-terminal tag was truncated in order to avoid irrelevant motions. Normal modes were computed on one structure and for each mode, we computed the RMSD of each model fitted onto the alternative structure. The first 6 modes corresponding to self rotations and translations applied to the whole system, were not taken into account further. The lowest RMSD value (1.029) was found to correspond to normal mode number 7, exhibiting a small torsion and a closure of the two domains. It clearly corresponds to a clamp motion, also known as a “Venus flytrap” motion, folding the two domains onto each other using the flexibility of the hinge region [32].

### Analysis of the CA3427 putative binding site

**Figure 2:**
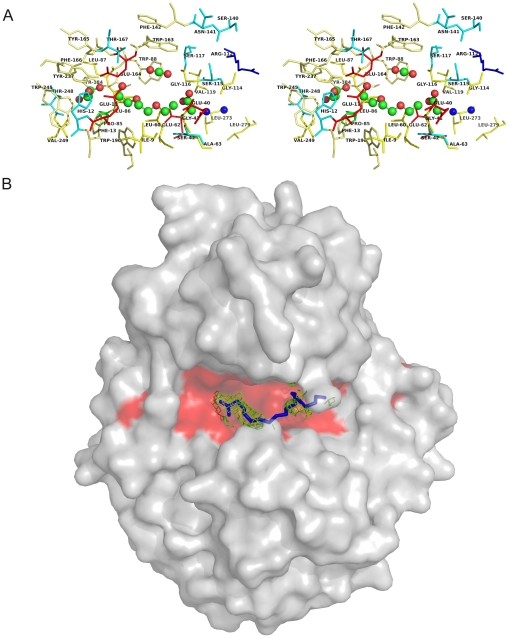
CA3427 putative binding site. A) Stereo view of the binding site with non polar residues and tyrosine in yellow, polar residues in light blue, acidic residues in red and proline in green sticks. Carbon dioxide, glycerol and PEG molecules are in ball and sticks with green carbon and red oxygen atoms. Two water molecules are represented as blue spheres. (B) Surface representation of the CA3427 structure with a modeled C18 acyl chain (blue ball and stick) fitted in the Fo-Fc electron density map (green) computed on the open conformation structure (1.5σ). The conserved residues in the predicted binding site are colored in red.

The two CA3427 structures exhibit extra electron density within the groove between the two domains, suggesting the localization of a ligand binding site. One region of extra density is common to both structure, and can be interpreted as a glycerol molecule (present in the crystallization medium). The other one, only showing in the open conformation, was interpreted as acetates, a PEG fragment (also present in the crystallization medium) and a carbon dioxide molecule. Although these molecules present in the crystallization medium are probably not the functional CA3427 ligands, we used them to identify the putative binding site consisting of the residues less than 5 Å apart from the co-crystallized molecules (Fig. 2A, Tables 2 and 3). Except for a small cluster of strictly conserved polar residues (Glu 11, His 12, Glu 164 and Thr 167) located at the PEG/Glycerol interface, this putative binding site is mainly hydrophobic (Fig. 2A). If we take into account the water molecules (2048, 2049) filling the remaining space of the groove between the two domains, the hypothetical binding site can be extended to include 4 supplementary conserved hydrophobic residues (G114, V119, L273, L279). This putative binding site could accommodate extended hydrophobic molecules such as long acyl chains (>C18) or carotenoids (Fig. 2B). We noticed that the position of the conserved histidine (H11), glutamate (E164) and threonine (T167) residues are not consistent with the usual geometry of catalytic triads in hydrolases. The precise function of the CA3427 protein thus remains to be determined.

**Table 2:** Putative ligand-protein interactions.

LI	LN	AL		RI	RN	D	LI	LN	AL		RI	RN	D
309	CA	CA	-	97	ASP	**3.29**	315	ACY	O	-	44	ARG	**2.94**
310	CL	CL	-	71	GLY	**2.86**	315	ACY	OXT	-	37	LYS	**3.48**
			-	73	GLU	**3.84**				-	38	VAL	**3.83**
311	GOL	C3	-	154	GLY	**3.24**				-	40	GLU	**2.79**
311	GOL	O3	-	154	GLY	**2.83**				-	44	ARG	**2.83**
312	GOL	C1	-	13	PHE	**3.36**	316	ACY	C	-	60	LEU	**3.94**
			-	164	GLU	**3.57**				-	62	GLU	**3.92**
			-	166	PHE	**3.89**				-	116	GLY	**3.48**
			-	245	TRP	**3.52**	316	ACY	CH3	-	63	ALA	**3.77**
312	GOL	C2	-	87	LEU	**3.63**	316	ACY	O	-	116	GLY	**3.16**
			-	164	GLU	**3.37**	316	ACY	OXT	-	60	LEU	**3.65**
			-	166	PHE	**3.9**				-	62	GLU	**2.78**
312	GOL	C3	-	85	PRO	**3.34**				-	116	GLY	**3.29**
			-	86	LEU	**3.97**	317	ACY	C	-	252	ASN	**3.82**
			-	87	LEU	**3.35**	317	ACY	O	-	252	ASN	**2.93**
312	GOL	O1	-	12	HIS	**3.64**				-	254	ARG	**3.6**
			-	13	PHE	**3.73**	317	ACY	OXT	-	259	PRO	**3.75**
			-	164	GLU	**2.69**	318	ACY	C	-	52	ASN	**3.35**
			-	166	PHE	**3.18**	318	ACY	C	-	197	ASP	**2.98**
			-	245	TRP	**3.17**	318	ACY	CH3	-	197	ASP	**3.92**
312	GOL	O2	-	86	LEU	**3.31**	318	ACY	O	-	52	ASN	**2.66**
			-	87	LEU	**2.84**				-	197	ASP	**2.57**
			-	164	GLU	**2.71**	318	ACY	OXT	-	50	ASN	**3.43**
312	GOL	O3	-	85	PRO	**3.87**				-	52	ASN	**3.3**
			-	248	THR	**2.74**				-	197	ASP	**3.31**
313	AE3	C1	-	42	SER	**3.53**	2592	CO2	C	-	88	TRP	**3.61**
			-	60	LEU	**3.63**				-	163	TRP	**3.77**
313	AE3	C2	-	9	ILE	**3.71**	2592	CO2	O1	-	88	TRP	**3.99**
			-	41	GLY	**3.74**				-	163	TRP	**3.76**
			-	42	SER	**3.41**				-	164	GLU	**3.31**
			-	60	LEU	**3.83**	2592	CO2	O2	-	88	TRP	**3.63**
313	AE3	C3	-	9	ILE	**3.49**				-	163	TRP	**3.88**
			-	190	TRP	**3.53**							
313	AE3	C4	-	11	GLU	**3.7**	1307	GOL	C1	-	13	PHE	3.85
			-	190	TRP	**3.74**				-	87	LEU	3.97
313	AE3	C5	-	190	TRP	**3.37**				-	164	GLU	3.63
313	AE3	C6	-	12	HIS	**3.85**				-	245	TRP	3.95
			-	13	PHE	**3.88**	1307	GOL	C2	-	85	PRO	3.49
			-	164	GLU	**3.72**				-	87	LEU	3.95
313	AE3	O2	-	60	LEU	**3.98**				-	248	THR	3.75
313	AE3	O4	-	11	GLU	**3.83**	1307	GOL	C3	-	85	PRO	3.73
			-	164	GLU	**3.08**				-	87	LEU	3.44
			-	167	THR	**3.44**				-	248	THR	3.64
314	ACY	C	-	83	LYS	**3.92**	1307	GOL	O1	-	86	LEU	3.95
			-	264	THR	**3.92**				-	87	LEU	2.87
314	ACY	CH3	-	85	PRO	**3.79**				-	164	GLU	3.11
314	ACY	O	-	186	PRO	**3.67**	1307	GOL	O2	-	248	THR	2.89
			-	187	TRP	**3.65**				-	249	VAL	3.86
			-	188	SER	**3.79**	1307	GOL	O3	-	85	PRO	3.25
			-	264	THR	**3.23**				-	184	TYR	3.67
314	ACY	OXT	-	83	LYS	**3.44**				-	248	THR	3.76
315	ACY	C	-	37	LYS	**3.58**	1308	CA	CA	-	52	ASN	2.9
			-	40	GLU	**3.93**				-	197	ASP	3.05
			-	44	ARG	**3.33**	1309	CA	CA	-	71	GLY	2.91
315	ACY	CH3	-	37	LYS	**3.29**				-	73	GLU	3

List of the shortest interatomic distances (<4 Å) between each of the ligands and the putative binding site amino acids. The distances were computed using NCONT (CCP4). Distance values are in bold for 2X7P, for 2X7Q otherwise. LI: ligand residue number, LN: ligand residue name, AL: ligand atom name, RI: protein residue number, RN: protein residue name, D: shortest interatomic distance between the ligand and the protein residue.

**Table 3:** Average B factor values for the interpreted ligands.

PDB	LIGAND	B factor*	Occupancy
2X7P	CA 309	57.1	1
	CL 310	55.9	1
	GLYCEROL 311	32.4	1
	GLYCEROL 312	20.7	1
	PEG 313	28.1	1
	ACETYL 314	28.3	1
	ACETYL 315	30.9	1
	ACETYL 316	24.9	1
	ACETYL 317	24.3	1
	ACETYL 318	24.6	1
	CO2 2592	22.8	1
2X7Q	GLYCEROL 307	14.5	1
	CA 308	20.5	1
	CA 309	24.2	1

*computed using CCP4.

The differences between the two CA3427 crystal forms were also analyzed in greater detail by comparing each domain separately. Superimposition of the domain I (RMSD = 0.53 Å) revealed only one major side chain reorganization within the predicted binding site. When the PEG/acetates molecules are present in the structure, the E11-O^ε2^ forms a hydrogen bond with Y237-OH (distance 2.7 Å). Upon pointing outwards from the binding pocket, it clears the space needed to accommodate the ligand. In the second structure, the above distance becomes 4.5 Å and the E11 side chain now points towards the inner part of the cavity. The domain II superimposition of the two structures (RMSD = 0.51 Å) again revealed a single noticeable difference within the binding site. The R112 side chain points towards the inside of the groove in the unliganded structure and outwards in the liganded one. Finally, in the closed conformation (i.e. without PEG/acetates molecules), D236-O^δ1^ (Domain I) and K170-N^ζ^ (Domain II) are linked by a salt bridge (distance 3 Å) that is disrupted in the presence of ligand (distance 4.8 Å), thus opening the “Venus flytrap”.

### CA3427 exhibits a PBP fold

In search for hints about the biochemical function of the CA3427 protein, we compared the newly determined structures against those in the Protein Data Bank [33] using Dali [34], [35] and VAST [36], [37] through their online servers. The best matching structural homologs all correspond to Periplasmic Binding Proteins (PBP) with RMSD between 2.9 and 4 Å and very low sequence similarity (lower than 16% identical residues) with CA3427 (Table 4). All these matching proteins are of bacterial origin and members of the class II PBP-like fold family: two similar intertwined domains of 3 layers (α/β/α) each. The β part is a duplication of mixed beta-sheet of 5 strands ordered as 2-1-3-5-4 with strand 5 antiparallel to the others. This PBP structural module, associated with a large variety of functions [38], is found in prokaryotic and eukaryotic protein families, as well as in the soluble part of the eukaryotic ionotropic glutamate receptors [39]. Due to their functional versatility, PBP have been considered promising protein engineering targets for biotechnology and drug delivery applications [38], [39], [40].

**Table 4:** Closest structural homologs of 2X7P and 2X7Q (Dali server).

PDB ID	Z-score	RMSD (Å)	LALI	NRES	ID (%)	PDB DESCRIPTION
2X26	25.4/24.7	3.3/3.6	283/280	308	12/12	Periplasmic aliphatic sulphonates-binding
3IX1	24.8/26	3.1/2.8	276/279	301	16/15	N-formyl-4-amino-5-aminomethyl-2-methylpyrimidine
3E4R	23.3/25.5	3/3	274/277	291	14/13	Nitrate transport protein
2I48	21.9/23.1	3.3/3.4	286/286	399	10/10	Bicarbonate transporter

Left and right values correspond to 2X7P and 2X7Q, respectively. RMSD (root mean square deviation): average distance between the Cα backbone atoms of the superimposed proteins. LALI: total number of aligned residues. NRES: length of the homologous protein sequence. ID: percentage of identical residues within the optimal alignment.

**Figure 3:**
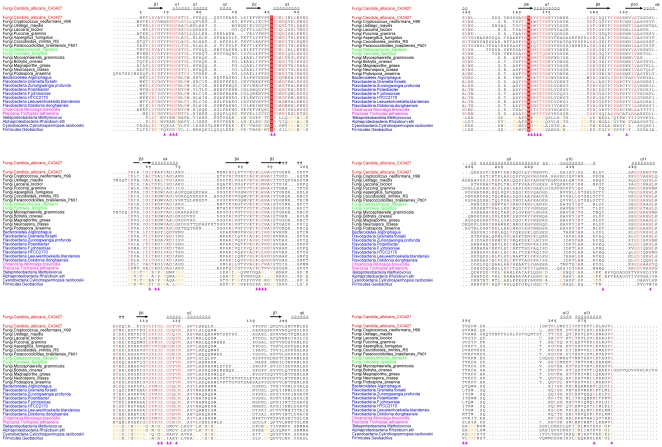
Multiple alignment of 29 selected sequences used for phylogeny. All fungi sequence names are in black except *C. albicans* (in red) *D. hanenii* and *Y. lipolitica* (in green). Other eukaryote sequence names are in magenta, bacterial sequence names are in light blue for Flavobacteria/Bacteroidetes and dark blue for the last 4 bacteria (outgroup). All the sequences are aligned together in one shot but similarity at each position is computed separately for the outgroup and the other sequences except for position 41 and 163 where strict conservation (white letters with red background) is observed. Red or orange letters in the alignment represent similarity (computed using a Risler matrix with a 0.9 threshold in ESPript) within each group. The secondary structure of CA3427 computed with DSSP, is also represented above the alignment. Purple triangles represent the binding site defined in the CA3427 structure. This alignment shows clearly the conservation of the binding site among different eukaryotes and Flavobacteria/Bacteroidetes but not in the outgroup.

The multiple sequence alignment of fungal proteins homologous to CA3427 highlights the conservation of the residues delimiting the groove (Fig. 3) further supporting its functional relevance. Moreover, the known binding sites of other PBP proteins are always located at the interface between the two domains as seen in the CA3427 structure. The corresponding conserved residues (Fig. 2A) thus define a new subfamily of PBP-related proteins, likely sharing a common ligand and a related function.

It hence appears that CA3427 was wrongly annotated (UNIPROT: Q59X88) as involved in the synthesis of thiamine, on the basis of its weak homology with the *Saccharomyces cerevisiae* ThI13 protein (UNIPROT: Q07748) (<20% identity over the full sequences length, blastp E value 0.29), since none of the binding site residues are conserved between the two sequences. In fact, the THI13 orthologue in *Candida albicans* corresponds to the Q5A3Y5 protein with which it shares 75% identity over 338 residues (E value: 10^−156^). The function of the new PBP-like family defined by CA3427 remains to be determined.

Interestingly, this new protein family is also well represented in flavobacteria and other members of the Cytophaga-Flexibacter-Bacteroides phylum of eubacteria to the exclusion of other prokaryotes. None of these proteins were functionally characterized. The binding site of CA3427 is strongly hydrophobic and can accommodate acyl chains much longer than C18 (Fig. 2B). It could thus participate in the detection, transport and/or processing of high molecular weight lipids (or carotenoids) in flavobacteria and fungi. Members of the CA3427 family are also present in two ancestral eukaryotes, the choanoflagellate *Monosiga brevicollis* and the placozoan *Trichoplax adhaerens*. These sequences share more than 30% identical residues with the CA3427 protein.

**Figure 4:**
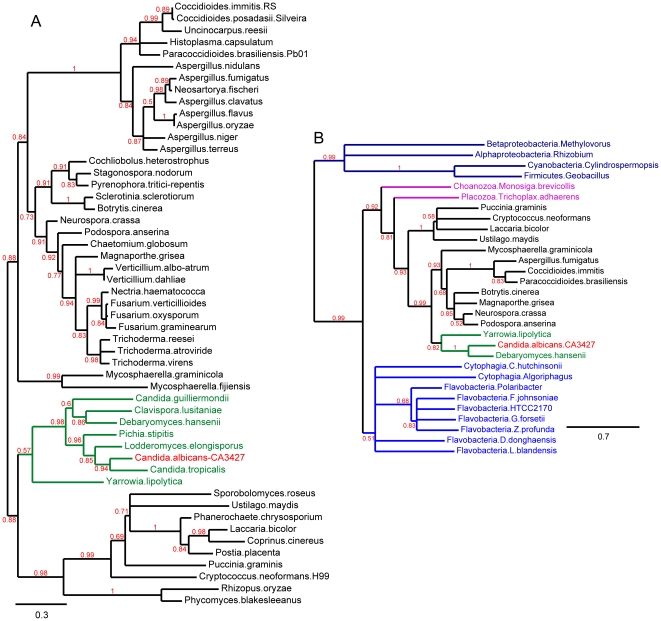
Phylogeny. **A**) **Phylogeny of the CA3427 homologs in Fungi.** The reference list of fungal species is from [23]. Multiple strains of the same species have been removed for clarity. The CA3427 sequence (in red in all trees) is from C. albicans strain SC5314. This unrooted tree was computed on the Phylogeny.fr web server [30], using the default option of the “advanced mode” w/o Gblocks. The final alignment includes 209 ungapped positions. Branch support estimates are indicated in red, and branches have been collapsed for values <50%. CA3427 homologs were found in all species except for saccharomycetales (in green), and cluster according to [23]. No species from the WGD clade (e.g. *Saccharomyces cerevisiae*) appear to possess a CA3427-like protein. **B**) **Evolutionary relationships between the bacterial PBPs and their eukaryotic CA3427-like homolog.** The phylogenetic analysis includes representative sequences from Cytophaga-Flavobacteria (light blue), Fungi (black and green) (as in Fig. 2), other eukaryotes (in magenta), and more remote bacterial sequences defining and outgroup (dark blue). This tree was computed on Phylogeny.fr web server [30], using the default option of the “advanced mode” without Gblocks. Branch support estimates are indicated in red, and branches have been collapsed for values <50%. The topology of this tree is consistent with the hypothesis that the original CA3427-like PBP was transferred into the eukaryotic gene pool from a cytophagia/flavobacteria into an ancestral opisthokont.

In order to investigate on the evolutionary origin of the C3427 protein family, we performed phylogenetic reconstructions. Figure 4A shows that the CA3427 phylogeny precisely follows the reference fungi classification [23]. A single inconsistency is the absence of a CA3427 homolog in *Saccharomyces cerevisiae*. In fact, all known species from the WGD (Whole Genome Duplication) saccharomycetales clade lack a CA3427 homolog, strongly suggesting that the loss of this gene coincided with the separation of the WGD clade from the CTG clade (the species that translate CTG as Serine instead of leucine) of saccharomycetales (e.g. Debaryomyces). In a more comprehensive phylogenetic reconstruction we included all members of the CA3427 family and, as an outgroup, representatives of bacterial PBP sequences not exhibiting the CA3427 binding site. The resulting tree (Figure 4B) strongly suggests that the eukaryotic and flavobacteria members of the CA3427 family originated from a common ancestral gene.

The presence of CA3427 homologs in the Bacteroides phylum of eubacteria strongly suggest that the PBP-like CA3427 protein has a very ancient bacterial origin. The divergence between the mainstream PBPs and the CA3427-like subfamily probably occurred early on the branch leading to the Bacteroidetes, after its separation from the branch leading to the other main groups of eubacteria (i.e. Proteobacteria, Planctomyces, Firmicutes).

**Figure 5:**
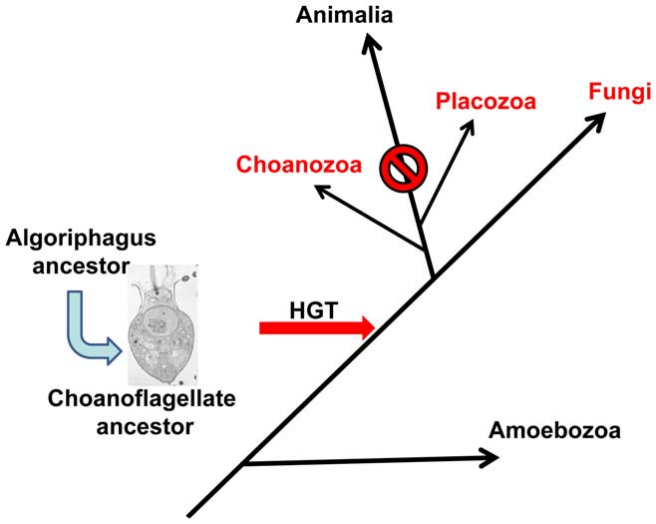
Parsimonious evolutionary scenario for the presence of CA3427-homologs in modern eukaryotes. A horizontal transfer (HGT) is proposed to have occurred from a bacteroidetes to an ancestral unicellular heterotrophic eukaryote prior to the divergence of the main branches leading to fungi and animalia. The branching of the various phyla is adapted from [42].

Finally, the surprising presence of a CA3427 homolog in the genome of the choanoflagellate *Monosiga brevicollis* as well as in the genome of placozoan *Trichoplax adhaerens*, the most basal invertebrate form, supports a scenario of horizontal transfer by which all eukaryotic CA3427 homologs originated from the above Bacteroidetes ancestor. Interestingly, Bacteroidetes species such as Algoriphagus are commonly found in association with modern choanoflagellates [41], thus providing opportunities for gene exchanges. The shared presence of CA3427 homologs in most fungi, the only sequenced choanoflagellate (closest unicellular relative of animals) and the only known placozoan (the closest multicellular relative of animals) strongly suggests that the transfer of the bacterial gene to an ancestral eukaryote occurred at the very basis of the opisthokont lineage, before the radiation of fungi [42] (Fig. 5). This gene was later lost in the branch leading to modern animals. Unexpectedly, the CA3427-like family of PBP thus provides a new marker to probe the early scheme of eukaryotic evolution [42].

We solved the 3-D structure of the Candida albicans CA3427 gene product, selected on the basis of its conservation among pathogenic fungi, and thus a potential target for new antifungal drugs. The structure of the protein unambiguously revealed a PBP fold, despite a low level of sequence similarity with previously known members of this family. In addition, the 3D structures allowed the precise delineation of a binding site, defined by highly conserved residues in the vicinity of co-crystallized ligands. The conformational change of the CA3427 protein upon ligand binding illustrates the venus fly trap motion already documented in other PBP structures [38], [39], [40], [43]. A phylogenetic analysis of the CA3427 protein family indicates that it originated in Bacteroidetes before being transferred to an ancestral eukaryote prior to the divergence between the fungi and animal lineages. The intriguing (albeit remote) possibility that the acquisition of this gene might be linked to the evolution towards multicellularity is a strong incentive for further functional studies. Furthermore, the conservation of this family of proteins in all pathogenic fungi coupled to its absence in animals makes it a good target for the design of new drugs against candidiasis and other diseases caused by fungi.

## Supporting Information

Movie S1 **Animated gif for the Morphing of the CA3427 venus flytrap motion.** The two structures were submitted to the Morph Server [44], the pictures were generated by pymol and concatenated to an animated gif with the ImageMagick convert function. The movie illustrates the venus flytrap motion of 2x7q leading to the 2x7p conformation. The strictly conserved residues in the groove are colored in red and residues with conserved properties are marked in yellow. Ligands are modeled as they appear in the opened conformation (2x7p). Carbon dioxide, glycerol and carboxyl molecules are in ball and sticks representation with green carbon and red oxygen atoms. Two water molecules are represented as blue spheres.
(GIF)
