# Supplementary figures and images for: The Conserved Candida albicans CA3427 Gene Product Defines a New Family of Proteins Exhibiting the Generic Periplasmic Binding Protein Structural Fold
Source: PLoS One. 2011 Apr 11;6(4):e18528. doi: 10.1371/journal.pone.0018528 (PMC3073944; doi:10.1371/journal.pone.0018528)

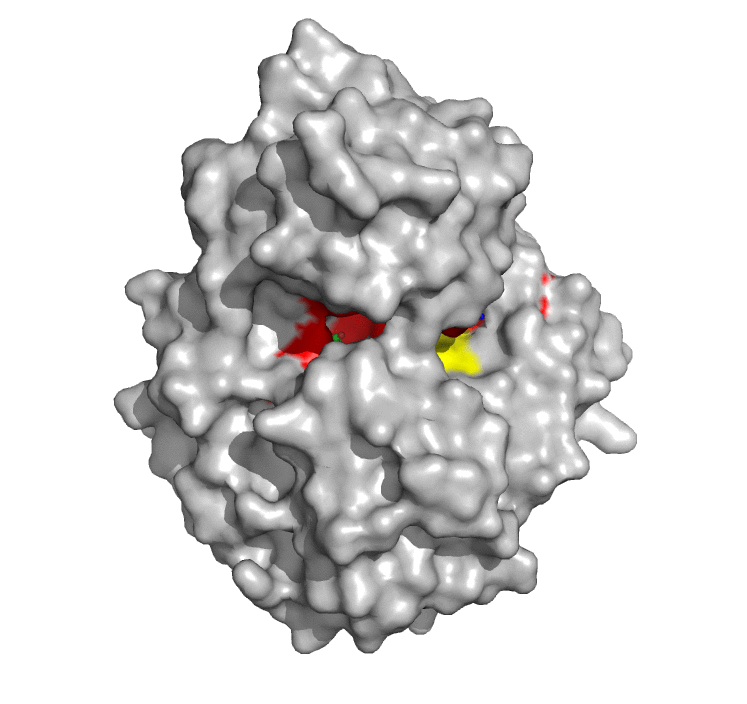

Supplement: Movie S1 — Animated gif for the Morphing of the CA3427 venus flytrap motion. The two structures were submitted to the Morph Server [44], the pictures were generated by pymol and concatenated to an animated gif with the ImageMagick convert function. The movie illustrates the venus flytrap motion of 2x7q leading to the 2x7p conformation. The strictly conserved residues in the groove are colored in red and residues with conserved properties are marked in yellow. Ligands are modeled as they appear in the opened conformation (2x7p). Carbon dioxide, glycerol and carboxyl molecules are in ball and sticks representation with green carbon and red oxygen atoms. Two water molecules are represented as blue spheres. (GIF) [file pone.0018528.s001.gif]
